# A challenging case of gastric outlet obstruction (Bouveret's syndrome): a case report

**DOI:** 10.1186/1752-1947-5-497

**Published:** 2011-10-04

**Authors:** Mahesh Gajendran, Thiruvengadam Muniraj, Andres Gelrud

**Affiliations:** 1 Department of General Internal Medicine, University of Pittsburgh Medical Center, 200 Lothrop Street, Suite 933W, Pittsburgh, PA 15213, USA; 2 Department of Gastroenterology, Hepatology and Nutrition, University of Pittsburgh Medical Center, 200 Lothrop Street M2, C Wing, Pittsburgh, PA 15213, USA

## Abstract

**Introduction:**

Bouveret's syndrome is a clinically distinct form of gallstone ileus caused by the formation of a fistula between the biliary tract and duodenum. This case reinforces the need for early recognition and treatment of Bouveret's syndrome, as it is associated with high morbidity and mortality rates.

**Case presentation:**

An 82-year-old Caucasian woman presented with signs and symptoms of small bowel obstruction. Her laboratory workup showed elevated alkaline phosphatase and amylase levels. Computed tomography of her abdomen revealed pneumobilia, a choledochoduodenal fistula and a gallstone obstructing her distal duodenum. The impacted gallstone could not be extracted endoscopically, so our patient underwent open enterolithotomy successfully. However, the postoperative course was complicated by myocardial infarction, respiratory failure and disseminated intravascular coagulation. She died 22 days after surgery, secondary to cardiopulmonary arrest.

**Conclusion:**

This case clearly highlights the considerable morbidity and mortality associated with Bouveret's syndrome.

## Introduction

Bouveret's syndrome is defined as a cholecystoduodenal or choledochoduodenal fistula with the passage of a gallstone into the duodenum or pylorus leading to gastric outlet obstruction [1]. There have been very few case reports about this syndrome published in the last 100 years because of its rarity. It is associated with high morbidity and mortality rates. With the availability of computed tomography (CT) scans, earlier diagnosis and better management of these cases are possible. Our patient had a typical presentation of the disease but ended up having multiple postoperative complications.

## Case presentation

An 82-year-old Caucasian woman, with a history of hypertension, depression, hypothyroidism, dyslipidemia, carotid endarterectomy and coronary artery disease, presented with a four-day history of nausea, bilious vomiting and epigastric pain radiating to her left scapula. Her home medications included sertraline, atenolol, calcitriol, levothyroxine, tolterodine, omeprazole, aspirin and atorvastatin. She denied smoking, use of alcohol or drug abuse. On examination, she appeared lethargic but not in acute distress. She was afebrile and had a blood pressure of 157/64 mmHg. Examination of her abdomen revealed abdominal distension, epigastric tenderness, tympanic sounds on percussion and decreased bowel sounds. Initial laboratory results were as follows: hemoglobin 11.4 g/dL, white blood cell count 6.2 × 10^9^/L, platelets 115 × 10^9^/L, creatinine 3.2 mg/dL (normal: < 1 mg/dL), blood urea nitrogen 30 mg/dL, amylase 710 U/L (normal: 30 to 110 U/L), lipase 133 U/L (normal: 22 to 51 U/L), albumin 3.1 g/dL, serum alkaline phosphatase 146 U/L (normal: 35 to 100 U/L) with normal levels of alanine transaminase (ALT), aspartate aminotransferase (AST) and total bilirubin.

**Figure 1:**
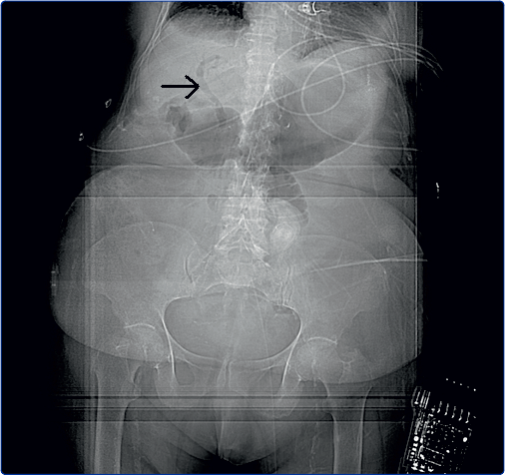
**CT scan of the patient's abdomen showing pneumobilia and a choledochoduodenal fistula**.

**Figure 2:**
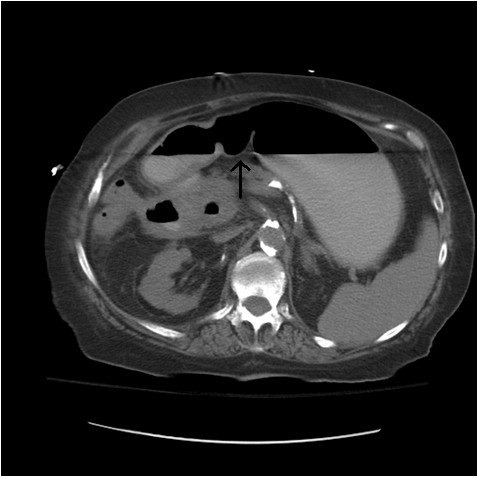
**CT scan of the patient's abdomen showing gastric and duodenal dilatation (gallstone ileus)**.

**Figure 3:**
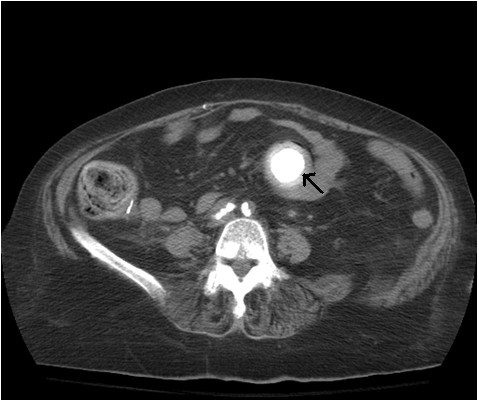
**CT scan showing a gallstone that is completely obstructing the patient's duodenum**.

**Figure 4:**
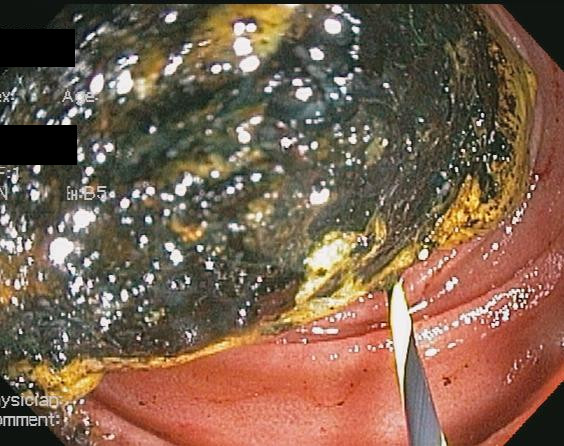
**Endoscopic view of the gallstone obstructing the patient's duodenum**.

A CT scan of her abdomen and pelvis with contrast (Figure 1) showed pneumobilia with a choledochoduodenal fistula (common bile duct and second part of her duodenum), significant wall thickening of the second portion of her duodenum and a large 3.6 cm gallstone obstructing her distal duodenum (Figures 2 and 3). Her stomach and proximal duodenum were dilated with decompression of the distal small and large bowel loops. These findings were consistent with gallstone ileus. In addition there was diffuse mesenteric stranding present throughout her abdomen without bowel wall thickening. An upper gastrointestinal (GI) endoscopy showed 1L of bilious fluid in her stomach with an impacted gallstone that could not be extracted with endoscopy (Figure 4). Our patient underwent an open jejunal enterolithotomy for gallstone removal without cholecystectomy. Also, a right hemicolectomy and ileotransverse colonic anastomosis were performed because of an ischemic ascending colon found intraoperatively. Pathology results revealed a gallstone and colonic mucosal ischemic changes. The postoperative course was complicated by a non-ST elevation myocardial infarction, pulmonary edema leading to respiratory failure requiring mechanical ventilation and disseminated intravascular coagulation manifesting as hemoperitoneum. Over the course of her hospital stay, her total bilirubin level increased up to 35 mg/dL with the direct bilirubin level being 19.8 mg/dL. Our patient had an international normalized ratio of 2.6 on postoperative day 22. Her AST and ALT levels were elevated at 203 U/L and 65 U/L, respectively, but her alkaline phosphatase level was normal. An abdominal ultrasonogram did not show any biliary dilatation. Our patient died 22 days after surgery secondary to cardiopulmonary arrest.

## Discussion

Bouveret's syndrome is a clinically distinct form of gallstone ileus (accounting for 1% to 3%), typically involving the proximal small intestine, which was first described by Leon Bouveret in 1896 [2]. It has a mortality rate of 4.5% to 25%. The major risk factors for developing this syndrome include age greater than 70 years, female gender, gallstones larger than 2.5 cm and postsurgical altered GI anatomy [3]. The presentation is similar to that of small bowel obstruction (SBO). The first diagnostic test for suspected SBO would be an abdominal radiograph; however, the classic Rigler's triad (pneumobilia, SBO and gallstone) has been reported to be present in only 30% to 35% of cases, since most of the gallstones are radiolucent. Contrast-enhanced CT evaluation of acute SBO offers prompt and rapid diagnosis of gallstone ileus [4]. It has a high sensitivity (93%), specificity (100%) and accuracy (99%) according to Yu *et al*. [5]. The first line of treatment should be upper endoscopy with an attempt to retrieve the stone. However, the success rate of this procedure has been only approximately 30% to 40%. Other minimally invasive techniques, such as laser lithotripsy and extracorporeal shock wave lithotripsy, are useful in high-risk patients when it is prudent to avoid surgery. In most cases, patients end up having surgery, most commonly enterolithotomy with or without cholecystectomy and fistula repair [6].

## Conclusion

This case clearly illustrates the considerable morbidity and mortality associated with Bouveret's syndrome. Preoperatively, establishing the diagnosis is the challenge, whereas postoperatively the management of complications can be even more challenging.

## Abbreviations

ALT: alanine transaminase; AST: aspartate aminotransferase; CT: computed tomography; GI: gastrointestinal; SBO: small bowel obstruction.

## Consent

Written informed consent was obtained from the patient's daughter for publication of this case report and any accompanying images. A copy of the written consent is available for review by the Editor-in-Chief of this journal.

## Competing interests

The authors report no financial relationships or conflicts of interest regarding the content herein. All the radiologic and endoscopic images are original.

## Authors' contributions

MG, TM, and AG analyzed and interpreted the patient data regarding our patient's presentation. MG was instrumental in obtaining informed consent from our patient's next of kin and also in the preparation of the manuscript. All authors read and approved the final manuscript.
